# On a Dangerous Form of Jaundice

**Published:** 1845-12

**Authors:** 


### SELECTIONS

FROM

### AMERICAN AND FOREIGN JOURNALS.

---

*On a dangerous form of Jaundice.*—Dr. Corrigan thus describes a form of jaundice of not unfrequent occurrence, and which, if not judiciously treated in the first instance, is apt to prove fatal by coma and other symptoms depending upon the non-elimination of the bilious secretion:

"This form of jaundice is to be met with, generally, among the poorer classes of society, but it occurs, with too great frequency, among persons of the middle rank of life, who have, for the most part, led sedentary lives. We find it occurring in merchants, whose lives have been made uneasy by having met reverses in trade, or by engaging in unsuccessful speculations. We find it occurring in females, mourning over the loss of a husband or a parent, while we as frequently meet with it in persons whose histories present nothing tangible to account for the disease. It sets in suddenly; sometimes we find the patient jaundiced all over in 30 hours, and this state of discoloration may continue for three days, three weeks, or as many months as weeks. In this form of jaundice, the pulse is regular, the tongue clean, the skin cool, and the appetite, in general, is tolerably fair; and we very often find, throughout the disease, the patient lively and cheerful, and able to perform the duties of life as well as ever. The only evidence of deranged health being, the jaundiced countenance, the white stools, and the urine loaded with bile. During this state of little, or no derangement, from the natural standard of health, if the slightest tendency to head affection, such as delirium or coma, should set in, no effort of your art can save the patient. All is at an end; for the records of medicine do not present a single case of recovery from the situation which I have just now described. I know that, in works on practice of medicine, you find this disease either not noticed at all, or if it is, merely as one of no importance. The most minute *post-mortem* investigations discover nothing whatever faulty, either in the brain, liver, gall-bladder, stomach, or intestines. With regard to its pathology, we are completely in the dark, but though I cannot give you any information on this head, yet as a set off to this want of pathological knowledge on my side, I think that I can give you what you will prefer to this, namely, an unerring cure for the affection in question, whenever it shall present itself to you in your future practice.

Having seen many cases of this disease unavailingly treated in town by blisters and leeches to the side, by the exhibition of purgatives, alkalies, and mercurials; knowing many such cases which were deemed incurable here, that afterwards were cured by country quacks by means of nauseous medicines; struck with this fact, and reasoning from the effects on the stomach of the quack medicines which had been given for its cure, I determined to try the effect of emetics in it. The event was completely successful, and I can assure you, that in an ample experience of four or five years since I first adopted this line of treatment, I have not had a single failure. In general, I am not wont to speak sanguinely of any remedies but those on which I can place implicit reliance, and the present occasion gives me an opportunity of speaking most favorably of the medicine which I have just been recommending to you. For the cure of this disease, it will be quite unnecessary for you to prescribe mercury, or any other medicine, save an emetic of ℨss. ipecacuanha every second day until the jaundice disappears. This frequently occurs after the action of the second emetic; even it will not be necessary for you to have recourse to purgatives, as the ipecacuanha in most instances relaxes the bowels; but if you do order purgatives, let them be of such a sort as will not color the fæces—so that you may perceive if the bile is again being propelled into the intestines. Of the medicines not liable to this objection, the best you can select are decoctum aloës compositum, and magnesia usta. By carrying into operation in your future practice, the routine which I have just sketched out for you, I am confident you will be able to add to mine, your unqualified approbation of the benefit derivable from the use of emetics in this form of jaundice.—*Med. Times, Jan.* 25, 1845—*Ranking's Half-yearly Abs.*

